# Research on Dynamic Pig Counting Method Based on Improved YOLOv7 Combined with DeepSORT

**DOI:** 10.3390/ani14081227

**Published:** 2024-04-19

**Authors:** Xiaobao Shao, Chengcheng Liu, Zhixuan Zhou, Wenjing Xue, Guoye Zhang, Jianyu Liu, Hongwen Yan

**Affiliations:** 1 College of Information Science and Engineering, Shanxi Agricultural University, Jinzhong 030801, China; syhjjj@126.com (X.S.); lc18133769501@126.com (C.L.); zhouzhixuan1003@126.com (Z.Z.); sxauxuewenjing@126.com (W.X.); z19834546770@126.com (G.Z.); 2 Science & Technology Information and Strategy Research Center of Shanxi, Taiyuan 030024, China

**Keywords:** pig counting, YOLOv7, DeepSORT, attention mechanisms, dynamic counting, PConv

## Abstract

**Simple Summary:**

A pig inventory is an important part of realizing accurate and large-scale farming. Distinguishing from the current, mostly overhead scene or static map counting, this study proposes a video-based dynamic counting method through which pigs can be counted automatically and continuously in complex scenes. The improved model effectively reduces the counting error and improves the operation efficiency, which provides data and experimental references for the study of automated pig counting, and it also provides technical support to improve the management efficiency of pig farms, reducing the cost and promoting the intelligent transformation of the animal husbandry industry.

**Abstract:**

A pig inventory is a crucial component of achieving precise and large-scale farming. In complex pigsty environments, due to pigs’ stress reactions and frequent obstructions, it is challenging to count them accurately and automatically. This difficulty contrasts with most current deep learning studies, which rely on overhead views or static images for counting. This research proposes a video-based dynamic counting method, combining YOLOv7 with DeepSORT. By utilizing the YOLOv7 network structure and optimizing the second and third 3 × 3 convolution operations in the head network ELAN-W with PConv, the model reduces the computational demand and improves the inference speed without sacrificing accuracy. To ensure that the network acquires accurate position perception information at oblique angles and extracts rich semantic information, we introduce the coordinate attention (CA) mechanism before the three re-referentialization paths (REPConv) in the head network, enhancing robustness in complex scenarios. Experimental results show that, compared to the original model, the improved model increases the mAP by 3.24, 0.05, and 1.00 percentage points for oblique, overhead, and all pig counting datasets, respectively, while reducing the computational cost by 3.6 GFLOPS. The enhanced YOLOv7 outperforms YOLOv5, YOLOv4, YOLOv3, Faster RCNN, and SSD in target detection with mAP improvements of 2.07, 5.20, 2.16, 7.05, and 19.73 percentage points, respectively. In dynamic counting experiments, the improved YOLOv7 combined with DeepSORT was tested on videos with total pig counts of 144, 201, 285, and 295, yielding errors of -3, -3, -4, and -26, respectively, with an average accuracy of 96.58% and an FPS of 22. This demonstrates the model’s capability of performing the real-time counting of pigs in various scenes, providing valuable data and references for automated pig counting research.

## 1. Introduction

China is the world’s largest pork producing and consuming country [1], with a large scale of pig farming. In 2023, the country’s pigs slaughtered totaled 726.62 million heads, an increase of 3.8% compared with 2022; the country’s pork production was 57.94 million tons, an increase of 4.6% compared with 2022, which was the highest level since 2015 [2]. In recent years, in order to meet the growing demand for pork, farms have generally adopted the model of large-scale farming and intensive management to expand the scale of farming. Pig counting is an important part of pig management, and its importance for pig farmers cannot be ignored. Traditional manual counting undoubtedly increases the labor cost of farming, and this process is time-consuming and inefficient, and it is prone to the problems of false reporting and omission [3]; especially in large-scale animal husbandry enterprises, these problems are more prominent. And in order to better manage pig farms, it is necessary to count the number of pigs in the unit in a timely manner so as to identify the disappearance or death of pigs and deal with such concerns on time to avoid the spread of diseases or a quality decline. However, traditional pig pen filling counting can only count pigs when they are in a pen, which cannot meet the requirement for real-time mastery of the number of pigs during the breeding process. Not only does sensor-based pig counting require the purchase and installation of sensor equipment, which is costly, but the sensor equipment may also be affected due to environmental factors, resulting in counting errors or equipment failure, which further affects the accuracy of counting. And wearable devices are invasive to pigs, and it is necessary to find a more suitable alternative to meet the counting requirements.

With the expansion of the scale of farming, pig management also needs more scientific and effective control. Nowadays, Chinese pig farms are gradually introducing advanced farming technologies and equipment, such as intelligent farming, digital management, and environmental control [4]. Through the application of intelligent monitoring, precise management, and other technical means, breeding efficiency is improved, the cost is reduced, breeding quality is ensured, and the sustainable development of the breeding industry is ensured. Computer vision technology provides a non-contact, low-cost counting method. Traditional computer vision counts by extracting features such as color, shape, and contour in an image, which in turn are counted using traditional ML algorithms such as k-means, support vector machines, and random forest algorithms [5,6]. Among them, Pandit et al. [7] proposed a silkworm egg counting method that utilizes grayscale images and contrast enhancement techniques for preprocessing and then employs thresholding and morphological operations to determine counts. However, this type of method mainly relies on morphological features such as color, shape, and contour for feature extraction, with a poor generalization ability [8], and the improper selection of feature equations or thresholds can cause fluctuations in performance. In complex scenarios, the method faces difficulty in producing satisfactory results.

In recent years, spurred by the rapid advancement of deep learning technology, deep learning-based techniques have been able to automatically extract features; among these techniques, convolutional neural networks have shown a strong feature extraction capability [9], and deep learning algorithms, such as the single shot multiBox detector (SSD) [10], EfficientDet [11], and you only look once (YOLO) [12], not only offer high accuracy and strong generalization ability but also offer a fast operation speed, and they have achieved better performance in practical applications. Therefore, a large number of researchers have applied deep learning algorithms to the field of agricultural counting, and significant results have been achieved in practical applications such as wheat counting [13,14], fruit yield estimation [15], and livestock inventory [16,17]. In the field of pig counting, Rong Wang et al. [18] proposed a high-density herd pig counting model based on the SOLOv2 algorithm, which integrates multi-scale feature pyramid and second-generation deformable convolution, and it reduces the consumption of computational resources and occupancy by optimizing the model structure so that the model can achieve the accurate counting of high-density herds with a segmentation accuracy of 96.7%. Tian et al. [19] accurately estimated the number of pigs in a whole image by modifying the counting convolutional neural network model with the ResNeXt structure and mapping the image features to the density map, which achieved an average absolute error of 1.67 with a real dataset. Feng et al. [20] proposed a pig counting and localization method for overhead shot images using a density map estimation technique. The method designed an efficient neural network that utilized depth-wise separable SK blocks and hybrid-vit blocks to obtain a density map and calculate the number of pigs; the method achieved a mean absolute error (MAE) of 0.726 for pig counting, and its pig localization precision was as high as 88.26%, while the recall rate was 86.02%. The above method, relying on density maps, has some advantages in dense crowded scenarios, but its limitation is that it is unable to retain detailed individual information and lacks access to accurate location information for individual targets. As a result, the method loses the ability to deal with associated targets over a time span, and it is not applicable to continuous counting [21]. In order to accurately identify pig locations, Ju et al. [22] proposed a real-time segmentation method using the Kinect depth sensor for the problem of the segmentation of pigs adhering to each other. A combination of YOLO and image processing was used to achieve the accurate separation of adherent pigs and improve their execution efficiency. Yang et al. [23] proposed an improved YOLOv5n pig inventory algorithm based on the construction of a multi-scene pig dataset and the introduction of the SE channel attention module, which improved the accuracy and robustness of the algorithm in complex occluded overlapping scenarios, and the algorithm’s MAE was 0.173. Hao et al. [24] proposed a novel pig detection and counting model based on YOLOv5, which combines the shuffle attention and Focal-CIoU loss; the shuffle attention module realizes multi-channel information fusion and enhances feature extraction performance, and the improved model achieves a high mAP and accuracy of 93.8% and 95.6% in pig detection and counting, respectively.

The above methods improve the robustness of the model in complex scenes by adding the attention mechanism, and they effectively improve pig counting accuracy, but due to the limited field of view of a single image and the fact that these methods do not realize continuous counting, they are not applicable to pig counting in a large pigsty. For this reason, many researchers have applied tracking techniques to the counting domain [25,26]. Cao et al. [27] achieved the dynamic counting of a small number of sheep by fusing the improved YOLOv5x and DeepSORT algorithms with the addition of the ECA mechanism, and they maintained a low error rate. Chen et al. [21] proposed a real-time automatic counting system based on a single fisheye camera, which achieved accurate counting through a bottom-up pig detection algorithm, a deep convolutional neural network keypoint detection and keypoint association method, an effective online tracking method, and a novel spatio-temporal response filtering method; however, the keypoint detection-based method relies heavily on the overhead shot detection angle, and it is not suitable for multitasking scenarios of counting. Jin et al. [28] designed an embedded pig counting system, which uses a lightweight object detector, Tiny-YOLOv4, and a tracking algorithm, DeepSORT, to realize real-time counting on embedded devices. Due to the limited performance of embedded devices, the lightweight detector faces difficulty in meeting the counting requirements of complex scenarios with occlusion and overlapping. Huang et al. [29] proposed an improved pig counting algorithm based on the more accurate YOLOv5x combined with the DeepSORT model. The algorithm improved the accuracy of pig target detection by embedding two SPP networks of different sizes in the YOLOv5x network and using SoftPool instead of MaxPool operations. In the video-based pig counting experiment, the correlation coefficient (R2) between the experimental results and the actual values reached 98.14%, but the algorithm can only count less than 10 pigs at a time to ensure accuracy, and the continuous counting needs to be divided into several times, which does not meet the requirements for dynamic counting. However, although the above methods realize continuous counting, this counting is based on the overhead angle, and no research has been done on the oblique shooting angle, which has a wide field of view and less obstruction, and which is conducive to the detection and identification of pigs. But the overhead angle is more limited in application scenarios, and the equipment needs to be deployed on top of a pigsty, which leads to more complicated deployment and maintenance.

Based on this, the current study proposes an improved you only look once version 7 (YOLOv7) model for detection and compiled a multi-scene pig counting dataset, including different barn sizes, different numbers of pigs in a group, different light intensities, different occlusion levels, and different shooting angles. For the occlusion and overlapping problem, this paper introduces the coordinate attention (CA) mechanism, which makes the model pay more attention to the information of different coordinate positions in the input image, and it helps improve the robustness and accuracy of the model in occluded, overlapping, and crowded scenes. Finally, the deep association metric SORT (DeepSORT) algorithm was used to realize the continuous dynamic counting of pigs under an arbitrary number, based on the above ideas. The work and innovations of this paper are summarized as follows:

1. This study constructed a pig counting data set in multiple scenarios, including different pig house sizes, numbers of pigs in groups, light intensities, degrees of occlusion, and shooting angles.
2. Aiming at the possible misdetection and omission problems in pig counting with complex scenes, this study introduces a CA mechanism to the head of the YOLOv7 model for optimization so that the model can dynamically adjust the degree of attention to different coordinate positions in an image.
3. This study proposes the P-ELAN-W module, which uses PConv to improve some convolution operations in the ELAN-W module in the head of the YOLOv7 model, reducing redundant calculations and memory accesses and maintaining good feature extraction capabilities.
4. Aiming at the problem of tracking ID jumping caused due to the phenomena of occlusion and crowding under slant shot angles, this study proposes a dynamic scanning counting method by combining the improved YOLOv7 and DeepSORT. Thus, it realizes dynamic counting under slant shot angles and effectively reduces the influence of tracking ID jumping on counting results.

## 2. Materials and Methods

**Figure 1:**
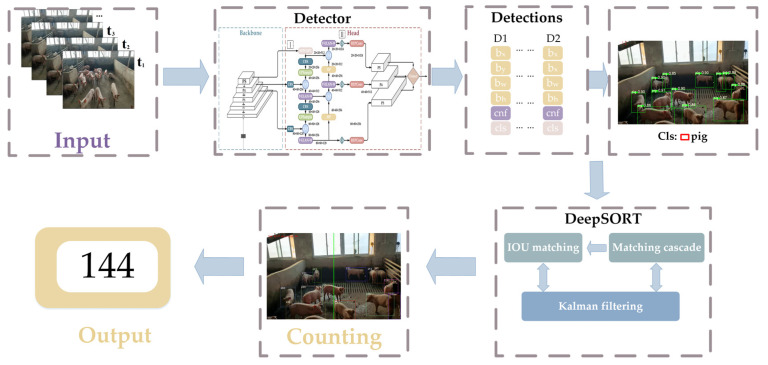
Flow chart of pig dynamic counting.

In this paper, a real-time pig dynamic counting system based on YOLOv7 and DeepSORT is proposed. This method distinguishes itself from solving the counting problem for static images, but it is targeted at realizing continuous counting for dynamic videos. The system is capable of detecting, tracking, and counting pigs within the camera’s field of view in real time, thus providing accurate and timely information to farmers. Figure 1 shows the overall framework of pig counting. It is divided into three steps: firstly, the current frame is detected using the improved YOLOv7 network, which accurately recognizes the position of the pig and the bounding box, and secondly, the detection results are fed into the DeepSORT algorithm, which associates the targets by combining the Kalman filter and the Hungarian algorithm. And at the same time, it extracts the target’s appearance features using the re-identification (ReID) network. This allows for associating and tracking the same pig and assigning the same ID between consecutive frames, and ultimately, the final dynamic counting is achieved via the contact of the counting line with the target frame.

### 2.1. Dataset Production

In order to realize the dynamic counting of pigs, this study divides the dataset production into four phases. The first phase is pig data collection to provide raw data. The second and third phases preprocess the raw data to construct the pig detection dataset and pig tracking dataset, respectively, to be used for the training and testing of the detection model and the ReID model. The fourth phase is the production of a dynamic counting video data set for the final dynamic counting experiment.

#### 2.1.1. Data Acquisition

This study utilized a combination of publicly available data and self-collected data. Most of the current studies collect data using the overhead shot angle, and there is a lack of publicly available multi-scene pig datasets. Therefore, in order to realize the task of counting pigs in complex environments, a multi-scenario pig counting dataset was constructed in this study, covering different scenarios of light intensity, shooting angles, pig density, degree of shading, and barn size.

**Figure 2:**
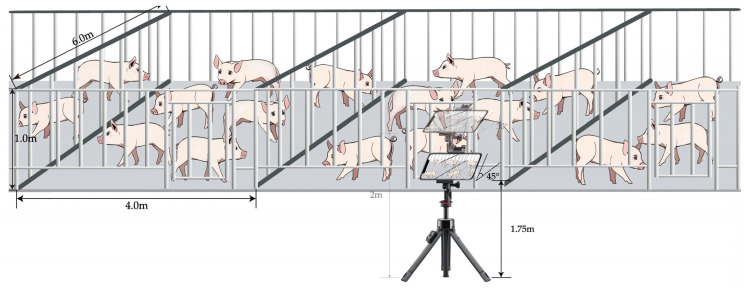
Schematic diagram of data acquisition method.

The data collection was divided into two parts: one part came from the pig inventory data set published by iFlytek [30]. This data set contains 700 images of pigs, mainly JPG format images taken from an overhead shot. In order to make up for the singleness of iFlytek data, another part of the data was obtained through manual field photography. In this study, the pig data were collected in late April and mid-May 2023, and the data were collected at two pig farms. The first pig farm was Lingbi Wen’s Pig Farm, Suzhou City, Anhui Province, with pen unit specifications of 4.0 m × 6.0 m, about 20 pigs set up in each unit, and a pig feeding density of about 1.2 m^2^; the other pig farm was Muyuan No. 14 Pig Farm, Si County, Suzhou City, Anhui Province, with pen unit specifications of 2.7 m × 6.0 m, about 18 pigs set up in each unit, and a pig feeding density of about 0.9 m^2^. During the acquisition process, the iPhone 14 cell phone was fixed on a tripod with a pulley, the height of the tripod was 1.75 m and 2 m, and the downward tilting angle of the filming equipment was 45 degrees and 30 degrees, respectively. In order to ensure the continuity of the data, each pig pen was captured in motion by slowly moving the pulleys and saved as a video. Figure 2 demonstrates the data collection method by taking Lingbi Wen’s Pig Farm, Suzhou City, Anhui Province, as an example. It should be noted that Figure 2 is only a reference display of the data acquisition method, which should be set reasonably according to the size of the pig farm, as well as the economic strength and management conditions of the pig farm. In the end, total data of 10 videos were collected, with an average length of 4 min. The frame rate of the videos is 30 FPS, and the resolution is 1920 × 1080.

#### 2.1.2. Data Pre-Processing in the Target Detection Phase

**Figure 3:**
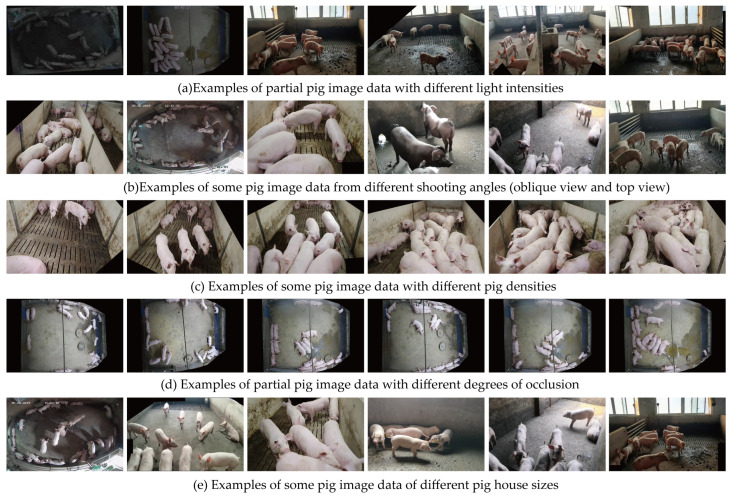
Example of original multi-scene partial pig data.

In order to obtain a better model input, the following operations were performed on the captured video: Firstly, the video was cropped every 20 frames to obtain 1800 images with a resolution of 1920 × 1080, which were saved in JPG format, data cleaning was performed manually to eliminate noisy data such as ghosting, blurring, and non-targeted objects, and 800 images were retained in the end. At the same time, the screened images were masked to cover some pigs in other pens. The manually collected data were slant shot datasets, which were fused with the public overhead shot datasets of iFlytek to obtain a 1500 multi-scene pig counting raw dataset, as shown in Figure 3.

**Figure 4:**
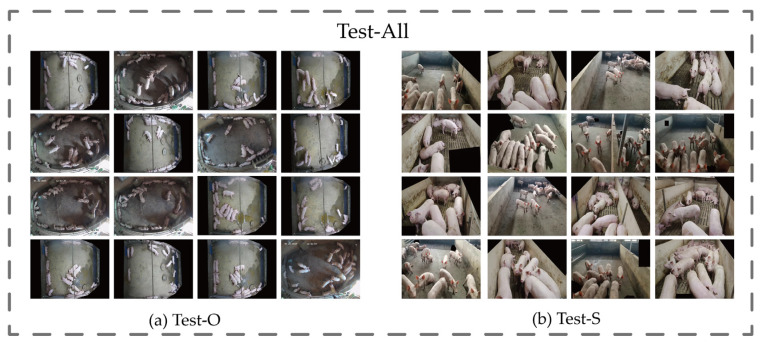
Test set data display chart.

The data were divided into a training set, a validation set, and a test set, respectively, at the ratio of 6:2:2 via a Python script in order to make sure that the ratio of slant shot images and overhead shot images was the same in each set, so the division was done using the slant shot datasets and the overhead shot test datasets, respectively. And finally, we obtained the training set of 972 sheets, a validation set of 264 sheets, and a test set of 264 sheets, of which the test set was divided into the slant shot test dataset (Test-S) and the overhead shot test dataset (Test-O), which totaled 133 and 131 sheets, respectively, as shown in Figure 4.

In order to improve the robustness and generalization ability of the model, as well as to ensure the veracity of the model with the validation and test sets, only the training set data were subjected to enhancement operations in this study. Six data enhancement operations, rotating, panning, changing brightness, adding noise, scaling, and flipping horizontally, were used to randomly enhance the data of some images in the training set, thus expanding the training dataset to 1441 images. After the above processing, a total of 1969 images were obtained, and the data were labeled using the LabelImg tool. Only the “pig” label was used in the labeling process, and the label file was saved in xml format. The pig dataset is shown in Table 1.

#### 2.1.3. Data Pre-Processing for the Target Tracking Phase

**Figure 5:**
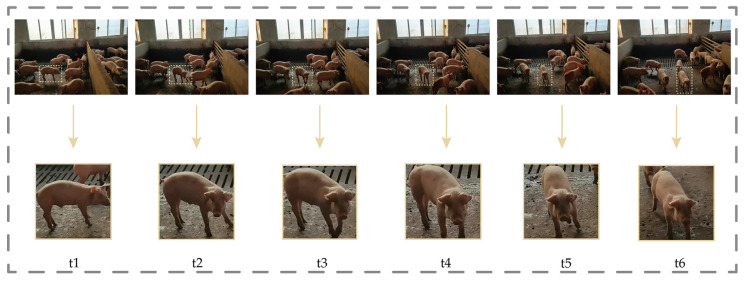
Schematic diagram of ReID data processing. t1–t6 respectively represent pictures of the same pig at different times.

Due to the lack of publicly available pig tracking datasets, this study constructed its own pig tracking dataset. As shown in Figure 5, firstly, three video sequences were selected as the dataset from the 10 captured pig videos. Each video was intercepted at 30-frame intervals with consecutive images as samples. The LabelImg tool was used to annotate the location of the same pig in multiple consecutive images, and a total of 50 pig trajectories were annotated with an average of 15 frames of images for each pig. Based on the labeled location information, pigs were cropped from the original images using a Python script, and images of the same pig were placed in the same folder. To ensure the quality and clarity of the data, the generated data were manually cleaned to remove blurred and heavily occluded images. Finally, 690 images were obtained with an average of 13 trajectory images per pig. The dataset was divided into a training set and a validation set, and the first image of each pig’s trajectory was selected as the validation set, while the remaining images were used as the training set for subsequent ReID model training and testing.

#### 2.1.4. Pre-Processing of Video Data in the Counting Phase

Four videos from the captured video data were used as test videos for the counting phase, named video-144, video-201, video-285, and video-294, respectively. The number after “video-” represents the total number of pigs in that video, and this value was counted frame by frame by three people to ensure its accuracy.

### 2.2. YOLOv7 Model Optimization Method

#### 2.2.1. The YOLOv7 Network Structure

YOLOv7 has received much attention as one of the most competitive target detection algorithms and model architectures currently available. Experiments have demonstrated that YOLOv7 outperforms all known object detectors in terms of speed and accuracy in the range of 5 FPS to 160 FPS [31]. In the dynamic counting task studied in this paper, the tracking algorithm DeepSORT will take up a certain amount of computational resources, and if the target detection part consumes too many resources, it will affect the overall operational efficiency. In order to achieve a balance between accuracy and speed, this study proposes preferring the YOLOv7 target detection model to construct the dynamic counting system.

**Figure 6:**
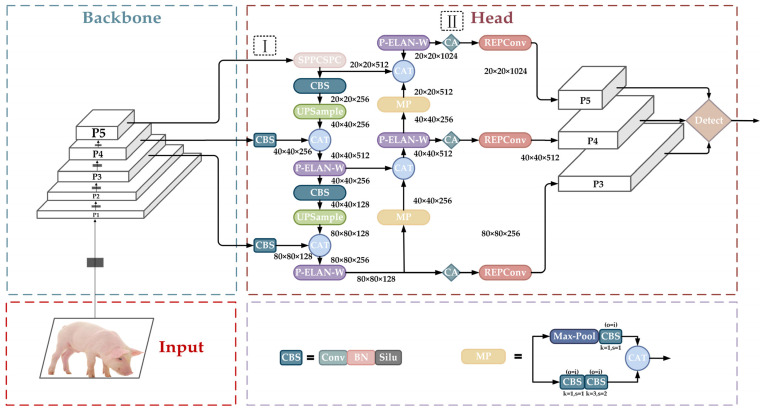
Improved YOLOv7 network structure diagram. P1, P2, P3, P4 and P5 represent different levels of the feature pyramid. I and II represent the two locations where the attention mechanism is added.

The network architecture of YOLOv7 [32] can be divided into three parts: the input layer, the backbone layer, and the head layer. The network architecture of YOLOv7 is shown in Figure 6.

The input layer is the entry point of the model, and it is responsible for pre-processing the input image and passing the pre-processed image to the backbone layer.

**Figure 7:**
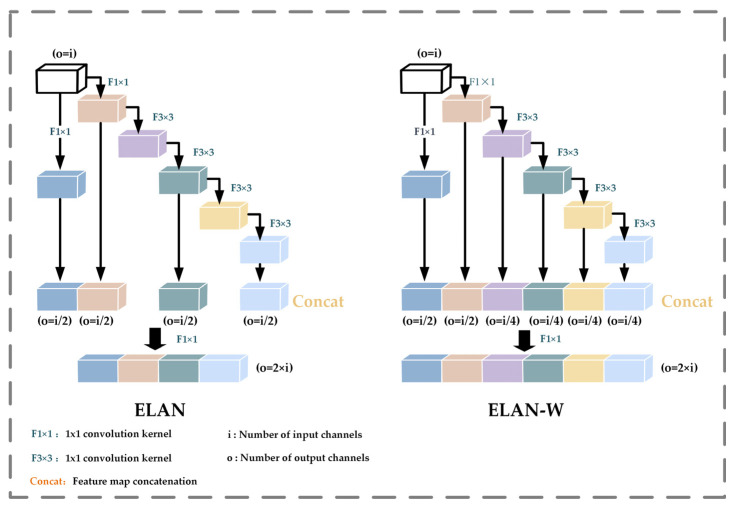
Structure of YOLOv7 backbone part ELAN module and head part ELAN-W.

The backbone layer is the core component of the YOLOv7 model, and its main function is to extract the target information features of three different sizes. Its most important feature is that it adopts the highly efficient ELAN [33] network architecture to extract rich feature information from the input image. ELAN is designed to solve the problem of the deterioration of the overall accuracy caused due to the expansion of the model size and to solve the problem of loss and over-inflation of information propagation by controlling the connecting paths of different lengths to learn and converge the network efficiently. The structure diagram of ELAN is shown in Figure 7.

The ELAN module consists of two branches, the short path, which varies the number of channels through 1 × 1 convolution for the fast propagation of gradients while enhancing the model’s understanding and robustness to inputs, and the long path, which retains more layers of features and extracts features through 1 × 1 convolution and four 3 × 3 convolutions, which are merged into a final feature representation. Finally, these four features are merged together to form the final feature extraction result. Depending on the number of outputs in the second branch, it is divided into ELAN and ELAN-W modules. The ELAN module comprises four branches for fusion, with the number of output channels doubled, primarily utilized during the feature extraction phase in the backbone. Meanwhile, the ELAN-W module features six fusion branches, also doubling the number of output channels compared to before. In addition, the backbone layer also includes the MP module, which combines the maximum pooling and convolutional block downsampling of the feature maps to reduce the computational burden and extend the perceptual range of the model.

**Figure 8:**
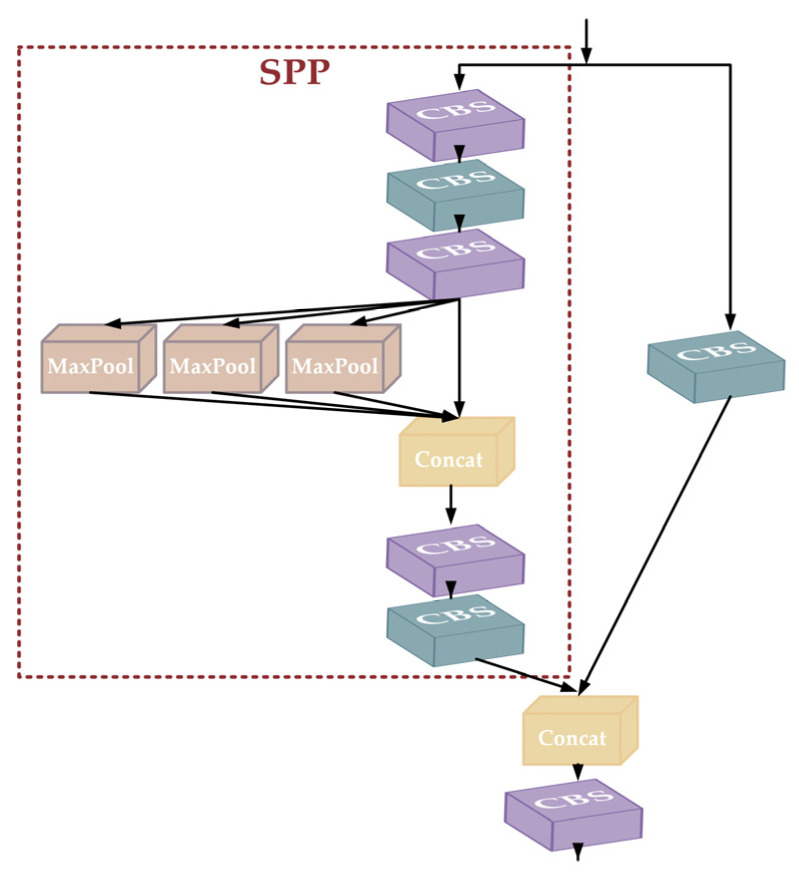
Structural diagram of SPCSPC.

The head part is designed using the strategy of the SPPCSPC module, which incorporates the two structures of SPP and CSP to enhance the feature characterization capability. The SPPCSPC structure is shown in Figure 8, where the SPP module processes the input feature maps through the three maximum pooling layers of different sizes, and these processes are carried out in parallel to retain spatial information at different scales. The CSP structure further enhances the learning capability of the model, which maintains the efficiency of forward propagation by dividing the feature maps into two parts, which are processed individually and then fused by the Concat operation. This design takes into account both the multi-scale representation of features and computational efficiency.

Next, the feature maps fusing multi-scale features and semantic information are sent to the final head component. The head layer utilizes a series of convolutional layers to further process these feature maps and predicts the bounding box, class, and confidence level of the target using the Detector component.

#### 2.2.2. PConv Optimizes ELAN-W Module

**Figure 9:**
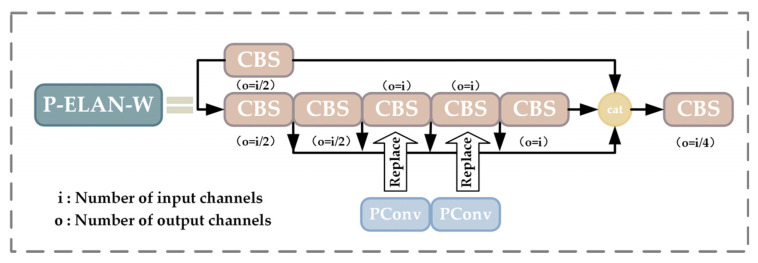
Schematic diagram of the improved P-ELAN-W module structure.

**Figure 10:**
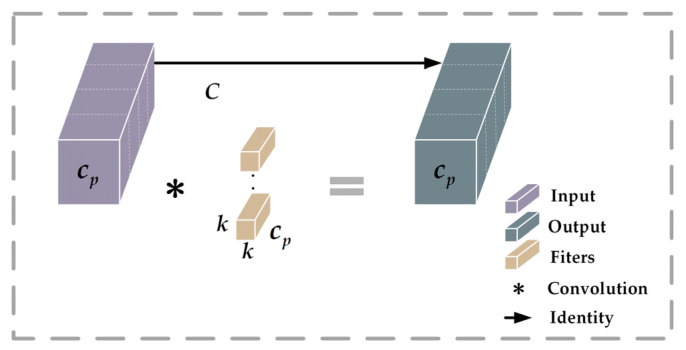
Schematic diagram of PConv structure.

In order to reduce the overall computational load of the model while ensuring the structural integrity of the ELAN-W network, this study proposes the P-ELAN-W module to optimize the two 3 × 3 convolution operations in the ELAN-W network through PConv. The improved ELAN-W can keep the original number of output channels unchanged. The P-ELAN-W network structure is shown in Figure 9:

PConv [34] is an efficient module designed to reduce the number of unnecessary parameters and computations when performing feature extraction, thus effectively improving the computational efficiency and performance of the network. Unlike conventional convolution, the PConv strategy applies regular convolution on selected channels of the input feature map while keeping the other channels unchanged. This treatment is particularly efficient for sequential memory reads because it treats neighboring channels as a valid representation of the entire feature map, and this treatment makes the input and output feature maps consistent in terms of the number of channels. The computational and memory access methods of PConv are significantly optimized compared to conventional convolution. The specific structure of PConv is shown in Figure 10:

In PConv, each convolution kernel operates on only a subset of the channels of the input feature map instead of computing all input channels as in conventional convolution. These selected channels are denoted as $c_{p}$, meaning that only a subset of the input channels ($c_{p}$) is used to generate the corresponding channels of the output feature map; this selective utilization of channels substantially decreases the number of parameters and computational load of the model.

The channel selection ratio (*r*) of PConv can be determined using Equation (1). This ratio indicates that the PConv network uses a subset of the original input channels for its operations. The *k* in Figure 10 indicates the size of the convolution kernel, while $c_{p}$ corresponds to the number of selected input channels.

(1) $$r=\frac{c_{p}}{c}$$

This computation saves significant computational resources over conventional convolution. Therefore, after the introduction of PConv, the design of P-ELAN-W can ensure the quality of the output feature map by maintaining the high efficiency of the network while maintaining a good feature extraction capability.

#### 2.2.3. Introducing CA Mechanism to Improve YOLOv7

**Figure 11:**
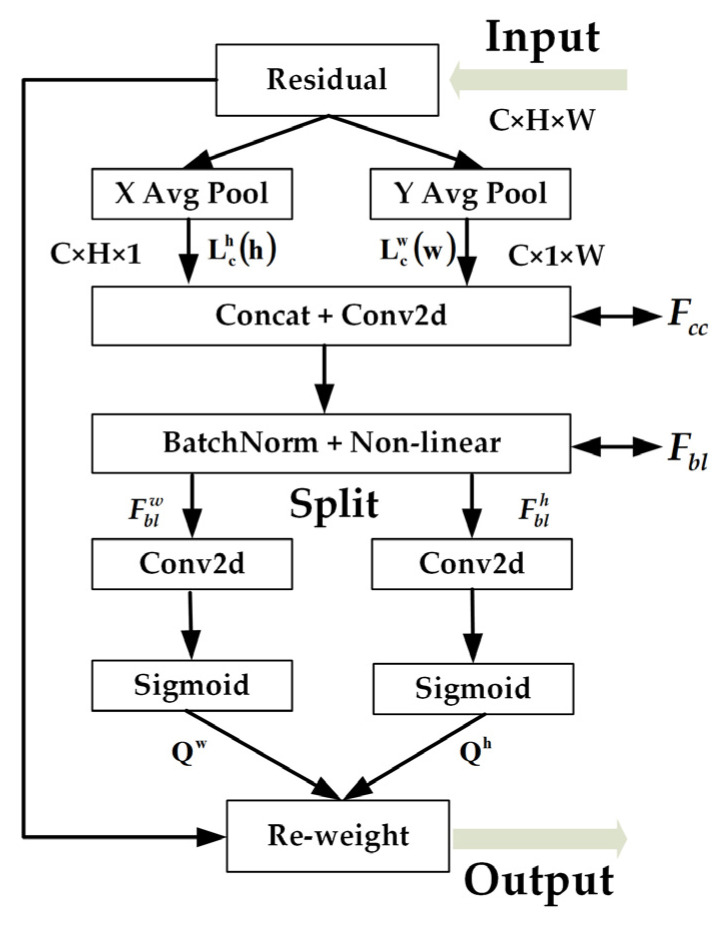
CA mechanism structure diagram.

Attention mechanisms are a set of mechanisms that derive weighting coefficients through autonomous learning via the network and “dynamically weight” them to emphasize regions of interest and suppress irrelevant background regions. SENet [35] is a mainstream attention mechanism that effectively establishes interdependencies between channels by simply compressing each 2D feature map interdependencies. CBAM [36] develops this idea further by introducing a large-size convolutional kernel combined with spatial information coding to further extract spatial features. These two approaches have been widely used in the past for various tasks in deep learning. However, the SE mechanism only focuses on the dependencies between channels, and it ignores the role of spatial features. On the other hand, CBAM introduces large-scale convolutional kernels to extract spatial features, but it fails to address long-range dependencies. Considering the need for lightweight network design, Hou et al. [37] proposed a novel attention mechanism, CA, which not only embeds position information into channel attention but also considers channel information and orientation-related position information to more fully utilize spatial features and solve the long-range dependency problem. Furthermore, the CA mechanism is also flexible and lightweight, allowing for easy integration into the network’s core modules. The diagram illustrating the CA mechanism is presented in Figure 11.

Firstly, it enhances its representation using the coordinate information in the input feature map, as shown in Figure 11; the input feature map has C channels with height H and width W. The input feature map is labeled a 3D tensor with dimensions of C × H × W. In order to capture the global context information along the height and width directions, respectively, the input feature map is processed via two average pooling operations, “XAvgPool” and “YAvgPool”, which aim to aggregate the information of the feature map in the spatial dimensions of W and H, respectively. These two operations output feature maps with dimensions of C × H × 1 and C × 1 × W, which represent the feature distribution information in the horizontal and vertical directions, respectively. The output of the Cth channel in terms of height and width is shown below:

(2) $$L_{c}^{h}\left(h\right)=\frac{1}{W}\underset{0\leq i\leq W}{\sum }l_{c}\left(h,i\right)$$

(3) $$L_{c}^{w}\left(h\right)=\frac{1}{H}\underset{0\leq j\leq H}{\sum }l_{c}\left(j,w\right)$$

In Equations (2) and (3), $l_{c}\left(h,i\right)$ is the value in row *H*, column *i*, of channel *C*, $l_{c}\left(j,w\right)$ is the value in row *j* of column *W* of channel *C*, $L_{c}^{h}\left(h\right)$ is the output along the horizontal direction, and $L_{c}^{w}\left(h\right)$ is the output along the horizontal direction.

Since the original features are aggregated in one dimension, the two feature maps need to be transformed in order to make them contain information from other dimensions. The feature tensor is concatenated and convolved via $F_{\mathrm{cc}}\;$to make the information in both directions interact with each other. Then, the feature mapping $F_{\mathrm{bl}}$ is obtained via a calculation to achieve the encoding of spatial information in the horizontal and vertical directions as follows:

(4) $$F_{\mathrm{bl}}=\delta \left(BN\left(F_{\mathrm{cc}}\right)\right)$$

where $F_{\mathrm{cc}}$ denotes the result after splicing along the spatial channel, δ is the nonlinear activation function, and BN denotes the batch normalization operation.

$F_{\mathrm{bl}}$ is split into two independent tensors, $F_{\mathrm{bl}}^{h}$ and $F_{\mathrm{bl}}^{w}$, along the spatial dimension, and it undergoes a convolutional transformation and an activation function that can make $F_{\mathrm{bl}}^{h}$ and $F_{\mathrm{bl}}^{w}$ have the same number of channels as the input feature map. Finally, $Q^{h}$ and $Q^{w}$ are obtained as attention weights. The specific formula is as follows:

(5) $$Q^{h}=\sigma \left(f_{h}\left(F_{\mathrm{bl}}^{h}\right)\right)$$

(6) $$Q^{w}=\sigma \left(f_{w}\left(F_{\mathrm{bl}}^{w}\right)\right)$$

In Equations (5) and (6), $f_{h}$ and $f_{w}$ are 1 × 1 convolutions, and σ denotes sigmoid activation functions.

In order to efficiently learn the pig features without significantly increasing the memory overhead and network depth, this study inserts the CA mechanism into the three reparameterized paths of the YOLOv7 network model, as shown in Figure 6, where the CA mechanism is added between P-ELAN-W and REPConv.

### 2.3. DeepSORT Algorithm

**Figure 12:**
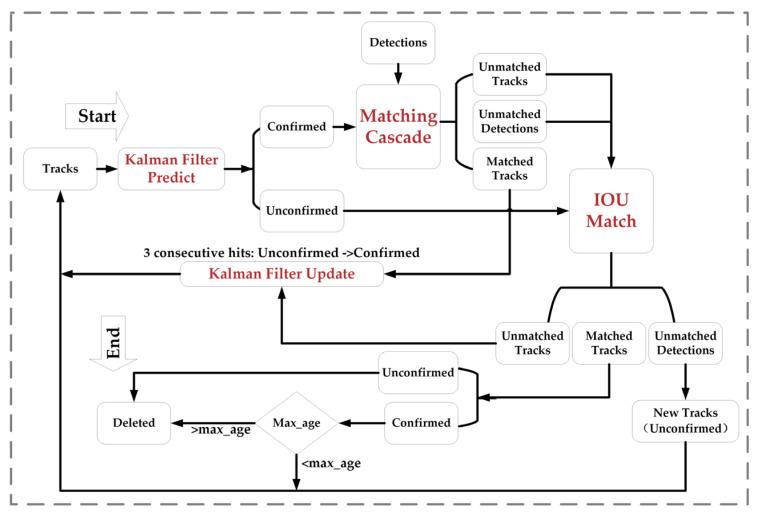
DeepSORT algorithm flow chart. Note: this figure is based on Figure 12 in [27].

DeepSORT [38] is a target tracking algorithm developed on the basis of SORT. It uses a convolutional neural network to extract the feature vectors of the target and uses metrics such as the Euclidean distance or cosine similarity to calculate the similarity between different targets in order to improve the accuracy and robustness of target tracking. DeepSORT mainly consists of two components: the Kalman filter and the Hungarian algorithm. The Kalman filter is utilized to predict and update the state estimation of the target, while the Hungarian algorithm is employed to match and correlate the prediction results of the Kalman filter with the detection outcomes of the target detector. The workflow diagram of the DeepSORT algorithm is depicted in Figure 12.

Firstly, in the initialization phase, this study used YOLOv7 to detect the target in the video frame and extract the feature vector of the target. Then, the Kalman filter algorithm was needed to estimate the target state through two phases: prediction and updating. After the Kalman filter had predicted the target, the Hungarian algorithm was used to calculate the similarity score between the predicted target and the target detection result of the current frame, which was used to determine which targets were the same object. Meanwhile, the Mahalanobis and Cosine distances are usually represented in Equations (7) and (9), which were used to compute the similarity between the feature vectors of the predicted target and those of the detected target to be used as elements of the cost matrix of the Hungarian algorithm to find the best target match.

(7) $$d^{\left(1\right)}\left(i,j\right)={\left(d_{j}-y_{i}\right)}^{T}S_{i}^{-1}\left(d_{j}-y_{i}\right)$$

where $y_{i}$ denotes the mean position of the trajectory, $S_{i}$ denotes the standard deviation of the trajectory, $d_{j}$ denotes the *j*th detection frame, and $\left(y_{i,}S_{i}\right)$ denotes the projection of the distribution of the *i*th trajectory into the measurement space.

The detected targets and predicted tracking states had to be matched. Typically, DeepSORT sets a threshold $t^{\left(1\right)}$, as shown in Equation (8), and when the Mahalanobis distance of two targets is less than this threshold, they are regarded as the same object, and they are associated.

(8) $$b_{i,j}^{\left(1\right)}=l\left[d^{\left(1\right)}\left(i,j\right)\leq t^{\left(1\right)}\right]$$

In order to overcome the rough prediction of the image motion state via the Kalman filter algorithm and the effect of motion uncertainty and camera jittering on the Mahalanobis distance correlation method, DeepSORT introduces a second correlation method based on the cosine distance. The specific steps are as follows:

Firstly, for each detected target frame, $d_{j}$ its feature vector, $r_{i}$ is computed via the deep learning model. The feature vector, $r_{i}$ is normalized such that its length is $\left\|r_{i}\right\|$. In addition, for the *i*th tracking target, an appearance description feature set, $R_{k}={\left\{r_{k}^{\left(i\right)}\right\}}_{k=1}^{L_{k}}$, is constructed to store the latest $L_{k}$-related appearance descriptions of each trajectory, *k*. Next, the minimum cosine distance between the target frame, $d_{j}$, and the feature vector in the gallery of the *i*th tracker is computed. Finally, the minimum value among all cosine distances is selected as the correlation metric between the current detection result and the corresponding tracker. The cosine distance is calculated as follows:

(9) $$d^{\left(2\right)}\left(i,j\right)=min\left\{1-r_{j}^{T}r_{k}^{\left(i\right)}|r_{k}^{\left(i\right)}\in R_{i}\right\}$$

If the minimum cosine distance is less than a preset threshold, the current detection result is considered the same target as this tracker. This cosine distance-based correlation method can better cope with motion uncertainty and camera jittering, thus improving the accuracy and robustness of target tracking. DeepSORT combines the linear weighting of the above two metrics as the final metric, and the calculation formula is as follows:

(10) $$c_{i,j}=\alpha d^{\left(1\right)}\left(i,j\right)+\left(1-\alpha \right)d^{\left(2\right)}\left(i,j\right)$$

A true association is considered to be achieved when $c_{i,j}$ lies within the intersection of the above two metric thresholds.

Finally, based on the matching results and detection criteria, DeepSORT manages the trace. For example, a track that cannot be matched for several consecutive frames may be marked as Unconfirmed or Deleted. Newly detected targets are added to the track list as New Tracks (Unconfirmed). A track becomes a confirmed track only after multiple consecutive frames have been successfully matched.

### 2.4. Counting Strategies

In the video-based wheat counting method proposed by Wu et al. [39] DeepSORT was used to track the wheat ears, and the number of IDs assigned via tracking was used as the final counting result. This method achieved a high accuracy of 95–98% in their wheat ear counting task. However, this counting accuracy heavily depends on the tracking performance of the target. In the dynamic pig counting task, pigs run and may be occluded, which can easily cause the tracking target ID to jump, thus affecting the final counting result. Based on this, a scanning counting strategy is proposed in this paper.

**Figure 13:**
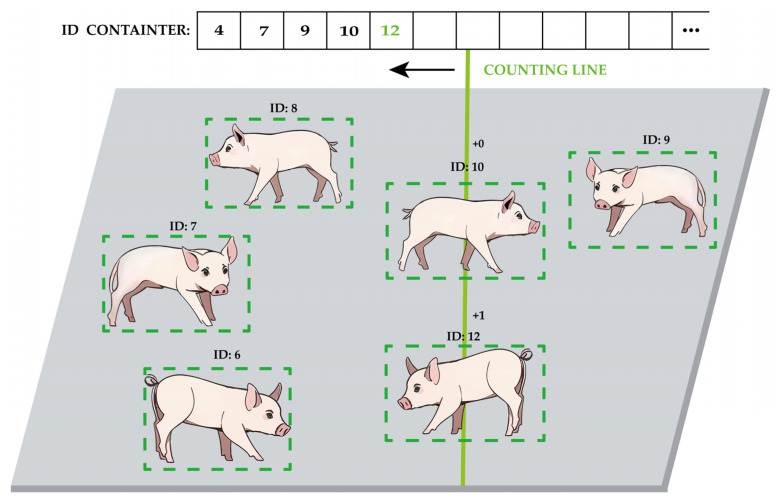
Schematic diagram of scan counting strategy. The green number 12 indicates the ID of the pig that was just counted.

As shown in Figure 13, in this strategy, a straight line is set in the center of the detection area as the counting line. Before counting, an ID container is first created for storing target IDs, and a unique ID number is assigned to each target via DeepSORT. Then, the pig and the counting line will move relative to each other, while the center of the detection frame will inevitably be passed by the counting line. When the detection frame is swept through, it is not counted directly, but the existence of the target is confirmed in the ID container. If the target has been recorded, it is ignored and not counted; if the target does not exist, it is counted, and its ID is recorded in the ID container.

### 2.5. Model Training

#### 2.5.1. Experimental Environment

The experiments in this study were conducted on a device running the Windows 10 Enterprise LTSC operating system. The device was equipped with a 12th Gen Intel(R) Core(TM) i5-12490F 3.00 GHz processor and 16 GB of RAM. Additionally, it was outfitted with an NVIDIA GeForce RTX 4060 graphics card with 8 GB of memory. The CUDA version used in the experiments was 10.2, the deep learning framework was PyTorch 1.7.0, and Python version 3.7 was employed. The parameter settings for YOLOv7 and DeepSORT during the experiments are presented in Table 2.

#### 2.5.2. Evaluation Metrics

The evaluation metrics in this study were divided into two parts: pig target detection and pig counting. For the pig target detection stage, the performance of the model was evaluated using the metrics precision, recall, *mAP*, $F_{1}$-Score, and FPS on the test data divided as per Section 2.1.2. Precision measures the proportion of targets predicted via the model to be pigs that are actually pigs, while recall measures the proportion of all actual pig targets that are correctly identified as pigs via the model. PR curves are plotted by recording precision and recall values, and *mAP* is the area enclosed within the PR curve. The $F_{1}$-Score is the reconciled average of precision and recall, and combining the performance of the two gives a more comprehensive assessment of the model’s performance. FPS refers to the number of frames per second that the model can process, and it can be used as a measure of the model’s operating speed. The equations for *Precision*, *Recall*, *mAP*, and$\;F_{1}$-Score are as follows.

(11) $$Precision=\frac{\mathrm{TP}}{\mathrm{TP}+\mathrm{FP}}$$

(12) $$\mathrm{Recall}=\frac{\mathrm{TP}}{\mathrm{TP}+\mathrm{FN}}$$

(13) $$F_{1}=2\cdot \frac{\mathrm{Precision}\cdot \mathrm{Recall}}{\mathrm{Precision}+\mathrm{Recall}}$$

(14) $$\mathrm{mAP}=\frac{1}{N}\sum_{k=i}^{\;N}\int_{0}^{\;1}P\left(R\right)\mathrm{dr}$$

where *TP* represents the number of correctly recognized pigs, *FP* represents the number of image regions that were incorrectly recognized as pigs, *FN* represents the number of pigs that were not recognized via the model, *P* is precision, *R* is recall, *P*(*R*) denotes the maximum precision when the recall is *r*, and *N* is the total number of categories, and in this paper, there is only one type of category, pigs.

For the video-based counting task, average_accuracy was used to evaluate the counting effect of this paper’s counting method on the test video described in Section 2.1.4.

(15) $$\mathrm{average}\_\mathrm{accuracy}=\frac{1}{m}\sum_{i}^{\;m}\frac{1-\left|a_{i}-\right.\left.b_{i}\right|}{a_{i}}$$

where m denotes the number of test samples, $a_{i}\;$denotes the number of real pigs in sample *i*, $b_{i}\;$ denotes the number of pigs detected via the model in sample *i*, $\left|a_{i}-\right.\left.b_{i}\right|\;$ denotes the number of errors, and average_accuracy denotes the average accuracy of counting in all the samples.

## 3. Results

### 3.1. Comparative Experiment on Pig Detection Performance of YOLOv7 Series Basic Models

The aim of this section is to compare the detection performance of the different models of the YOLOv7 family in order to provide a basis for selecting the most suitable model for the dynamic pig counting task. YOLOv7-tiny, YOLOv7, YOLOv7x, YOLOv7-w6, YOLOv7-d6, YOLOv7-e6, and YOLOv7-e6e were trained under the experimental environment and training parameters described in Section 2.5 using their respective pre-trained weights. Then, 264 images from the test set were used for testing. The attributes of the seven models and their monitoring performance are shown in Table 3.

As can be seen in Table 3, YOLOv7-tiny, as a lightweight model, has an image detection speed of 3.12 milliseconds per image and a model size of only 13 MB, which makes it suitable for embedded device deployment, but with lower accuracy. The YOLOv7 model size is moderate at 71.3 M, with up to 97.16% precision detected with the full dataset, Test-All. YOLOv7x is scaled in depth and width relative to YOLOv7, and on the full dataset, Test-All, mAP_0.5, mAP_0.5:0.95, and recall improved by 0.25, 0.21, and 0.48 percentage points, respectively, and the precision decreased by 0.19 percentage points, while the model size increased by 67.7 M. YOLOv7-w6 is optimized for cloud GPUs, but the results show that its performance does not improve on regular GPUs. YOLOv7-e6 and YOLOv7-d6 are scaled and tuned based on YOLOv7-w6; YOLOv7-e6 has the highest accuracy with the full test datasets, and the accuracy of YOLOv7-d6 is also better than that of YOLOv7, but the YOLOv7-d6 model size is 1480 M, which is not suitable for practical applications. YOLOv7-e6e is a model further optimized via YOLOv7-E6 using E-ELAN, which, despite this optimization, suffers from a slight performance degradation in the test datasets.

For the dynamic pig counting task in combination with DeepSORT, the accuracy and speed of the target detection phase are equally important. If the target detection produces a large number of false detections, it will lead to biased counting results. At the same time, a large model resulting in a slow detection speed will also seriously affect practical applications. YOLOv7 achieved a good balance between accuracy and speed in the experiments, so it was selected as the detection model for the dynamic counting experiments, and subsequent optimization experiments were carried out on this basis.

### 3.2. Improved YOLOv7 Model Detection Performance Experiment

#### 3.2.1. Model Detection Performance Experiment after Attention Mechanism Optimization

In order to verify the effects of different attention mechanisms and their addition locations on the model, the channel attention mechanism, CA, the stochastic attention mechanism shuffle attention (SA) [40], and the hybrid attention mechanism, CBAM, were added to YOLOv7 with two addition locations: add location I, the last layer of the backbone network, and add location II, between the ELAN-W module and the REPConv module, as shown in Table 4. In this experiment, YOLOv7 was used as the basic model, the resolution of the input image was 640 × 640, each model was trained for 100 epochs, and each model was tested for its performance under two viewpoint test sets, Test-O and Test-S.

From Table 4, it can be observed that the addition of attention mechanisms has a positive impact on the overall performance of the model without increasing its size. The enhancement of YOLOv7 is particularly significant after the addition of CA and CBAM mechanisms at position II. The model with added CA has a mAP of 94.54%, a recall of 94.44%, and a precision of 97.41% with the entire test dataset. Compared to the original model, these metrics improved by 0.97, 1.40, and 0.25 percentage points, respectively. On the other hand, the model with CBAM added has a mAP of 94.97%, a recall of 94.42%, and a precision of 96.93% with the entire test dataset. Compared to the original model, MAP and recall improved by 1.40 percentage points and 1.38 percentage points, respectively, while precision decreased slightly by 0.23 percentage points.

Comparing the different positions added via the attention mechanism revealed that the model improved the mAP, recall, and precision detected with the full test set by adding CA at position II compared to position I by 0.48, 0.74, and 0.17 percentage points, respectively; the addition of CBAM at position II compared to the addition of position I improved the MAP and recall by 0.40 and 0.16 percentage points and achieved a slight decrease of 0.39 percentage points in precision.

In terms of the model detection results for different angles of data, the base model YOLOv7 improved by 8.65, 7.53, and 4.25 percentage points the mAP, recall, and precision relative to the slant shot angles for the overhead shot angle. After adding the attentional mechanism, CA, SA, and CBAM were added in manner II and improved relative to the base model at slant shot angles by 3.59, 1.02, and 4.28 percentage points in the mAP, by 3.0, 0.99, and 4.23 white points in the recall, and by 3.85, 0.77, and 1.80 precision percentage points. However, in the overhead shot angle, the attention mechanism improved the model less, and it even decreased in some indicators, with the SA, CBAM, and CA added in manner II achieved decreases of 0.20, 0.06, and 0.99 percentage points in precision relative to the original model, of which the SA decreased by 0.54 percentage points in the mAP.

A comprehensive analysis revealed that the overhead shot angle is more conducive to the model detection of pigs because it offers a broader field of view with less mutual obstruction among pig groups. Conversely, the slant shot angles, due to their lower angle, are prone to clustering and obstruction, thereby affecting detection performance. The addition of attention mechanisms effectively improves the model’s performance at slant shot angles. Specifically, CA enhances the model’s perception of different locations by assigning different weights to each coordinate position in the input. In obstructed scenes, the CA attention module helps the model focus more on areas that may contain pigs. Meanwhile, adding attention mechanisms to the head network is more effective than adding them to the backbone.

#### 3.2.2. Visual Analysis of the Impact of Attention Mechanism on Model Detection Performance

**Figure 14:**
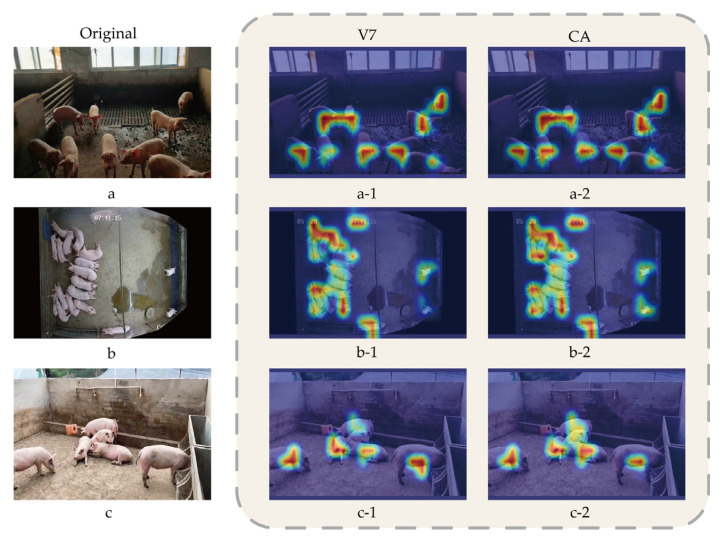
Heat map generated via YOLOv7 and YOLOv7+CA. **a**, **b**, and **c** represent the original images respectively, **a-1**, **b-1**, and **c-1** respectively represent the heat maps generated by the YOLOv7 model on **a**, **b**, and **c**, and **a-2**, **b-2**, and **c-2** represent respectively The heat map generated by the YOLOv7 model on **a**, **b**, **c** after adding the CA mechanism.

To intuitively demonstrate the areas of focus of the model and the role of attention mechanisms in guiding the network to key areas, this study utilized Grad-CAM [41] for visualization to present the decision-making process from the second layer to the final layer of the model. Grad-CAM generated heatmaps for YOLOv7 and YOLOv7 with the added CA mechanism, as shown in Figure 14.

In the heatmap, the intensity of colors represents the model’s confidence in detecting targets, with the red areas highlighting the key regions where the model predicts the target’s position. As shown in Figure 14, both models are capable of accurately distinguishing between targets and backgrounds. In Figure 14(a-1,a-2), the model’s focus is on the head region of the pig, indicating that the model has successfully learned the most distinctive features of the pig, which helps improve the accuracy of pig detection and reduces false detections. Additionally, after adding the CA mechanism, the colors in the heatmap become darker, indicating higher confidence. In the heatmap of group b, it can be seen from Figure 14(b-1) that YOLOv7 does not pay enough attention to the pig in the bottom right corner, which can easily lead to missed detections. However, after adding the CA mechanism, the attention to this pig is significantly enhanced. The heatmap of group c shows that, although YOLOv7’s attention points can distinguish targets from the background, some background areas are still included in the attention region. With the addition of the CA mechanism, the network can more accurately focus the attention points on the target pig.

The results of this experiment show that the introduction of CA makes the network’s focus on the critical region more explicit and further improves the model’s recognition accuracy with the target. This has a positive impact on optimizing the pig detection task.

### 3.3. Experiments on the Impact of Different IoU Thresholds on the Detection Performance of the Base and Improved Models

**Figure 15:**
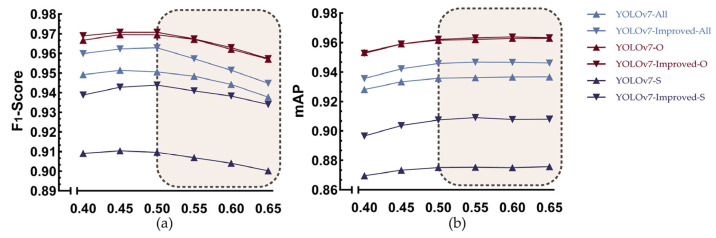
(**a**) The $F_{1}$-Score of YOLOv7 and YOLOv7-Improved detected with the Test-S, Test-O, and Test-All datasets under different IOU thresholds; (**b**) the mAP of YOLOv7 and YOLOv7-Improved detected with the Test-S, Test-O, and Test-All datasets under different IOU thresholds.

The IOU threshold is a criterion used in object detection to measure the degree of overlap between the detection box and the ground truth box. By adjusting the IOU threshold, the strictness of non-maximum suppression (NMS) can be controlled. In order to optimize the model’s performance and find the best threshold settings, this experiment optimized the ELAN-W module using PConv, introduced the CA mechanism into the head network, and named the improved YOLOv7 YOLOv7-Improved. Under IOU thresholds of 0.4, 0.45, 0.5, 0.55, 0.6, and 0.65, YOLOv7 and YOLOv7-Improved were tested with the Test-S, Test-O, and Test-All datasets in this paper. The experimental results are shown in Figure 15.

In Figure 15a, within the range of 0.4 to 0.65, all curves initially rise with the increase in the IOU threshold and then gradually start to decline, indicating that increasing the IOU threshold can improve the $F_{1}$-Score before reaching 0.5. In Figure 15b, the mAP value continues to increase with the increase in the IOU threshold, but the growth rate starts to slow down after the IOU threshold reaches 0.5. Meanwhile, it is observed that the improved YOLOv7 outperforms the original model with three test datasets, with the greatest improvement observed under the slant shot angles. In summary, the model performs best when the IOU threshold is 0.5, and the improved model demonstrates good generalization from different perspectives, indicating stability and strong adaptability to data from different angles.

### 3.4. Ablation Experiments

In this experiment, PConv was utilized to optimize ELAN-W, and the CA attention mechanism was introduced. Ablation experiments were conducted on the improved YOLOv7 model using the Test-S, Test-O, and Test-All datasets in this paper. The IOU threshold was set to 0.5, and the experimental results are presented in Table 5.

From Table 5, it can be observed that Model II is a lightweight P-ELAN-W optimized YOLOv7 model. Compared to the original model, it achieved an improvement of 1.49 percentage points in mAP and 1.25 percentage points in recall with the Test-S dataset. Meanwhile, the computational complexity decreased by 3.7 GFLOPS, although there was a slight decrease in precision. Model III introduced the CA attention mechanism to the head part of YOLOv7. Relative to the original model, it showed improvements of 3.59, 3.0, and 3.85 percentage points in mAP, recall, and precision, respectively, with the Test-S dataset. With the Test-All dataset, it achieved improvements of 0.97, 1.40, and 0.25 percentage points in mAP, recall, and precision, respectively. Model IV optimized YOLOv7 simultaneously with P-ELA-W and CA. Compared to the original model, it exhibited improvements of 3.24, 0.05, and 1.00 percentage points in mAP with the Test-S, Test-O, and Test-All datasets, respectively. In terms of recall, it achieved improvements of 2.54, 0.23, and 1.34 percentage points versus precision improvements of 4.39, 0, and 1.10 percentage points, with a decrease in computational complexity of 3.6 GFLOPS.

The comprehensive ablation test revealed that the use of PConv to improve ELAN-W can significantly reduce the amount of computation and, at the same time, improve the performance of the model under slant shot angles, and the addition of CA attention mechanism improves the model’s ability to perceive different locations. In the occlusion scenario, CA helps the model pay more attention to the area that may contain pigs, and the detection effect is again improved.

### 3.5. Comparison Experiment for Detection Performance of Optimized YOLOv7 Model and Other Models

This experiment compared the improved YOLOv7 model with other typical target detection algorithm models, Faster RCNN, YOLOv5, YOLOv4, YOLOv3, and SSD. The IOU threshold was set to 0.5, and the tests were conducted with the Test-All dataset of this paper, respectively, in order to prove the improved YOLOv7 model’s experimental scenario of this paper’s superiority, and the results are shown in Table 6.

From Table 6, it can be observed that YOLOv5 has a model size of 40.1 MB, making it more efficient and convenient for model deployment and mobile applications. YOLOv5 achieves a mAP of 92.5%, demonstrating good object detection accuracy. However, the recall is relatively low at 86.90%, indicating that there may be instances where targets are not detected. The SSD (mobilenetv2) model has a smaller size of only 14.3 M, and it achieves an FPS of 116.05. However, its performance in terms of mAP, recall, and precision is relatively average, and it may face limitations in detecting fine-grained objects. The Faster RCNN model has a larger model size of 521 M, with a higher recall rate but relatively lower precision. It may encounter some false positive detections.

Improved-YOLOv7 further enhances performance based on YOLOv7 with an increase of 1.0, 1.34, and 1.1 percentage points in mAP, recall, and precision, respectively, compared to YOLOv7. Compared to YOLOv5, it achieves increases of 2.07, 7.48, and 2.36 percentage points in mAP, recall, and precision, respectively. Compared to YOLOv4, it achieves increases of 5.20, 4.57, and 1.43 percentage points in mAP, recall, and precision, respectively. Compared to YOLOv3, it achieves increases of 2.16, 1.67, and 0.83 percentage points in mAP, recall, and precision, respectively. Compared to SSD, it achieves increases of 19.73, 18.20, and 3.85 percentage points in mAP, recall, and precision, respectively. Compared to Faster RCNN, it achieves increases of 7.05, 3.09, and 32.93 percentage points in mAP, recall, and precision, respectively.

In summary, YOLOv7 is at the forefront of object detection, offering high recall and accuracy and achieving a better balance between precision and recall. The improved YOLOv7 further enhances its performance, providing a reliable foundation for subsequent dynamic counting tasks.

### 3.6. Visualize the Detection Effect of the Model in Different Scenarios

For the detection effect of the algorithm, this experiment compared the improved YOLOv7 with the original YOLOv7 model in occluded, dim, and overhead shot situations.

**Figure 16:**
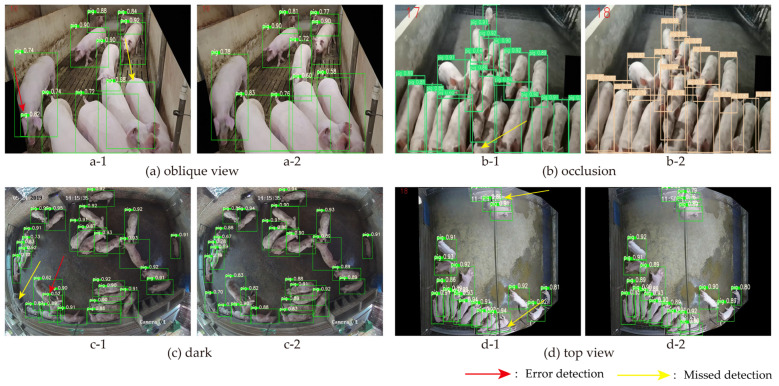
Actual detection effect of YOLOv7 and improved YOLOv7. **a-1**, **b-1**, **c-1**, and **d-1** represent the detection result images of the original YOLOv7 model, while **a-2**, **b-2**, **c-2**, and **d-2** represent the detection result images of the improved YOLOv7 model.

The detection results are shown in Figure 16, from which it can be seen that the original YOLOv7 has a large number of missed and misdetected cases. In Figure 16(a-1), a pig was missed, and the pig’s ear was mistakenly detected as the pig itself. In Figure 16(b-1), a high degree of crowding led to missed detection. Although Figure 16c,d show overhead angles, the dense pig population resulted in pig-to-pig sticking. The original YOLOv7 could not effectively distinguish between these adherent pigs, leading to missed and false detections.

The improved YOLOv7 showed significant improvement in detection performance, and the improved model can still accurately detect all pigs in crowded and pig sticking scenarios. As shown in Figure 16a,b in the crowded and occluded scenario, the detection accuracy for pigs is up to 90%. The highest accuracy for pig detection is up to 94% under dim conditions, as shown in Figure 16c.

### 3.7. ReID Model Training

**Figure 17:**
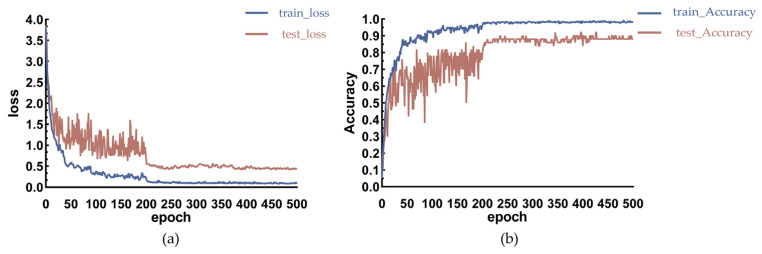
(**a**) denotes the loss value for ReID model training and testing, and (**b**) denotes the correctness rate for ReID model training and testing.

DeepSORT is mainly used for the tracking of pedestrians, while the original ReID [42] model is mainly applicable to the appearance re-identification of pedestrians, and it is not applicable to the re-identification of pigs. Therefore, in order to achieve the tracking of pigs, this experiment retrained the ReID model on the pig re-identification dataset in this paper in order to extract the appearance features of pigs to improve the tracking effect. The change curves of the loss value and accuracy of the ReID model during the training and testing process are shown in Figure 17.

As shown in Figure 17, the loss curve and accuracy curve gradually smooth out as the number of iterations of the model increases, indicating that the model gradually converges. After 200 rounds of iterations, the loss curve on the test set was stable at 0.407, and the test accuracy was stable at about 0.92. When the test accuracy was close to 1, it indicates that the model performs better in the re-identification of pigs. The re-identification accuracy of 0.92 fully meets the demand for dynamic counting in this paper.

### 3.8. Dynamic Pig Counting Experiment

**Figure 18:**
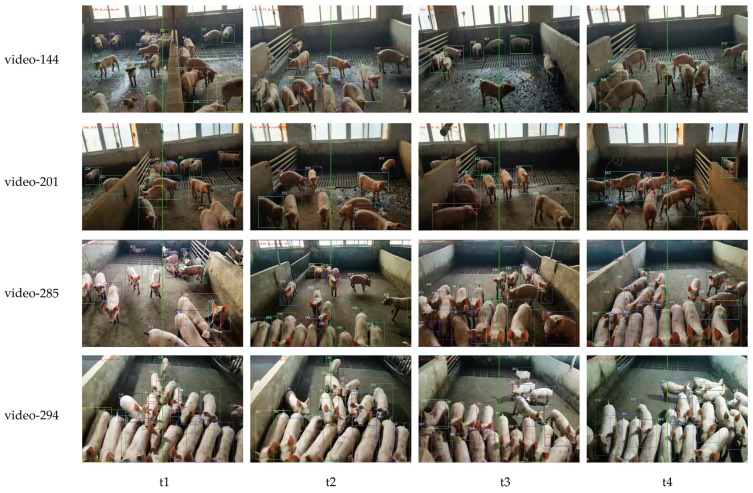
Graphs of counting effects at different moments for video-144, video-201, video-285, and video-294 test videos. t1, t2, t3, and t4 respectively represent four moments in a detection video.

The ameliorated YOLOv7-Improved was fused with DeepSORT to perform dynamic counting experiments with the video-144, video-201, video-285, and video-294 video test sets, and the counting process is shown in Figure 18.

As shown in Figure 18, the model can accurately detect a pig while assigning a tracking ID to it; with a counting line in the center of the figure, the target box scanned using the counting line will determine whether its ID is in the ID container. If not, it will be added to the ID container and counted, and vice versa without counting. The counting results are shown in Table 7:

As can be seen in Table 7, the first group used the experimental method of taking the total number of tracking ID assignments directly as the counting result. The experimental results show that the counting results of this counting method deviate from the actual results and exceed the actual number by a large amount. The average error of the original model is 144 heads, and the average error of the improved model is 129 heads; the average error was slightly reduced, but the counting effect is still not satisfactory. The average accuracy of the model before improvement is 41.25%, and the improved model is slightly relieved, but the counting effect is still unsatisfactory, with an average accuracy of 46.93%. The second group adopts the counting strategy proposed in Section 2.4 of this paper, and its counting effect is significantly improved. The error counts of the original model on the 4-video test set are −4, +5, +19, and −32, with an average accuracy of 94.33%. Meanwhile, the improved model has error counts of −3, +3, −4, and −26 with the same test set and an average accuracy of 96.58%.

The counting technique proposed in this paper effectively alleviates the dependence on the tracking effect. The results show that the counting effect is significantly improved, while the improved YOLOv7 is proven to have an enhancing effect on the final counting result.

## 4. Discussion

### 4.1. Analysis of Pig Detection Performance from Different Shooting Angles

Currently, most pig counting studies are conducted from an overhead shot angle, with few focusing on slant shot angles. In order to make up for the lack of research on counting from slant shot angles, we constructed a multi-scene pig counting dataset, which includes data from both overhead shot and slant shot angles. We tested the YOLOv7 model on both overhead shot and slant shot test datasets. The experimental results show that, compared to slant shot angles, YOLOv7 performs significantly better in terms of MAP, recall, and precision at overhead shot angles, with improvements of 8.65, 7.53, and 4.25 percentage points, respectively. The superior performance at overhead shot angles is likely due to the broader field of view and reduced occlusion among pig groups. Conversely, slant shot angles, with their lower perspectives, are prone to pig clustering and occlusion, thereby affecting detection performance. However, counting applications cannot be confined to overhead shot angles alone. Slant shot angles are more suitable for handheld devices in small pig farms. Therefore, this study did not overlook the application of slant shot angles but, rather, improved the model by adding a CA mechanism. The addition of the attention mechanism effectively enhances the model’s performance at slant shot angles. Specifically, the model’s performance after the incorporation of the CA attention mechanism improved by 3.59, 3.0, and 3.85 percentage points in terms of MAP, recall, and precision, respectively, compared to the base model. Visual analysis through heatmaps shows that the introduction of CA makes the network’s focus on critical areas more distinct, further enhancing the model’s accuracy in pig recognition and indicating that adding attention mechanisms has a positive impact on optimizing object detection tasks. Nonetheless, the performance of the CA-improved model for detection at overhead angles is still 5.04 and 4.79 percentage points higher in terms of mAP, recall, and precision compared to oblique camera angles.

### 4.2. Analysis of Target Detection Performance Using Different Optimization Methods

In this study, the performance of the YOLOv7 series models was first tested. Of them, YOLOv7 exhibited the most balanced performance in terms of model size and performance. With a size of 71.3 M, it achieved a MAP, a recall, and precision of 93.57%, 93.04%, and 97.16%, respectively, with the entire test set. At the same time, experiments found that YOLOv7x, YOLOv7-d6, and YOLOv7-e6e have more complex network structures but do not demonstrate stronger performance. This may be because large models typically require more data for training and parameter tuning to fully exploit their expressive capabilities. If the available training data are limited, large models may struggle to learn enough patterns and features from the data [43], thereby affecting their accuracy. Therefore, under specific task and data constraints, smaller models may be more suitable for achieving better performance.

In addition, this study lightened YOLOv7 by using PConv to replace part of the convolution in the ELAN-W module in HEAD, which decreased the model computation by 3.7 GFLOPS, while the model’s performance did not show a significant decrease. Aiming at the problems of overlapping and occlusion in the process of counting pigs in oblique shooting, the attention mechanism was used to improve YOLOv7, and the effects of the CA, SA, and CBAM attention mechanisms on the model detection performance at different additive positions were compared in the experiments of this paper. The experimental results show that adding the attention mechanism has a positive impact on the model performance in general without increasing the model size. Meanwhile, we found that the attention mechanism affects the model differently under different angles. In the research scenario of this paper, the key features in the image (the facial features of the pig) can be better emphasized under the oblique shooting angle, and the attention mechanism successfully captures these features and gives them higher weights. On the contrary, the model is not improved much after adding the attention mechanism under the top shot angle, probably because the distribution of image information under this angle is not conducive to the work of the attention mechanism, or the key features are hidden or weakened.

### 4.3. Adaptations and Limitations of Counting Methods

The DeepSORT-based dynamic counting task should flexibly adopt targeted counting strategies when dealing with different application scenarios. Let us take static targets such as wheat ears as an example [39]; although it is simple and easy to directly use the number of IDs assigned via DeepSORT as the counting result, the accuracy of this method is highly dependent on the tracking effect. In complex scenarios such as pig counting, experiments have shown that the frequent switching of tracking IDs and large counting errors are easily caused due to the occlusion and running behavior among pigs. In the scanning counting approach proposed in this paper, the core counting trigger condition occurs when the counting line is in contact with the center point of the target frame. Since this contact is usually brief, even if there is a jump in ID afterward, it will not affect the final counting result. At the same time, to ensure that repeated counts are not performed on the same pig, the method also records the IDs that have already been scanned. After practical testing, this method exhibits high counting accuracy when a pig is moving relatively slowly; e.g., counting errors of −3, −3, and −4 were recorded in the video-144, video-201, and video-285 videos, respectively. However, the counting errors of the video-295 test video were −3, −3, and −4 when a person was moving relatively slowly. In the video-295 test video, when a human approached the shot, the stress response of the pigs caused them to congregate and cover each other, which caused the detection frame to disappear briefly, and it could not be scanned using the counting line, leading to an increase in missed detections. In addition, there are cases where pigs that have already been recorded via the counting line re-pass the counting line, which may lead to double counting. Although DeepSORT is able to match pigs that have passed the counting line multiple times as the same pig and assign the same ID to them to avoid double counting, tracking is often ineffective when pigs are moving quickly and are obscured by other pigs in the middle of the counting line, which leads to the problem of double counting. Therefore, to ensure counting accuracy, this method should be chosen to count pigs when they are moving more gently or when they are relatively still. In the future, this research will further optimize and improve the tracking model and counting strategy to improve the stability and accuracy of counting in complex scenarios.

### 4.4. Future Application Scenarios and Research Directions

**Figure 19:**
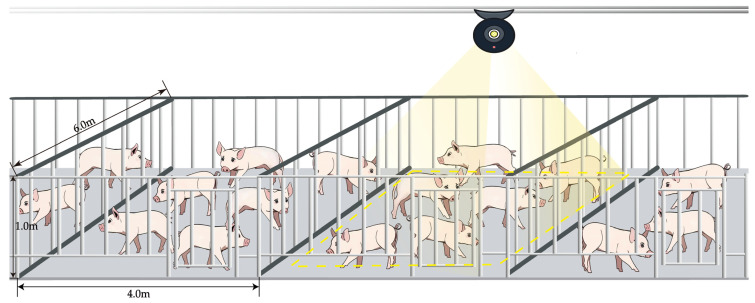
Schematic diagram of track camera detection.

The dynamic counting system in this study is capable of achieving accurate counting in a wide range of scenarios, including counting tasks from overhead and other angles. Different application strategies can be used for different sizes of farms:

1. For farming retailers, the number of farmed pigs is relatively small, with less occlusion, which is more suitable for handheld device counting. In future research, the handling model can be further lightened, and a counting application can be developed for use by retail farmers.
2. For large farms with a large number of pig pens and large sites, manual handheld counting is less efficient. The counting method under the top view angle can be adopted, as shown in Figure 19, where a track camera is installed above the pig house, and dynamic counting is realized by moving the camera. This approach can count without disturbing pigs, and it can effectively avoid obstruction, improve the counting accuracy, and at the same time reduce the labor cost of a farm.

## 5. Conclusions

This study aimed to realize the dynamic real-time counting of pigs in multiple scenarios. By comparing the training of various series of YOLOv7 models, the most suitable YOLOv7 model for this study was selected for experiments. On this basis, the YOLOv7 model was improved: firstly, using PCony, the second and third 3 × 3 convolution operations in the ELAN-W module of the head network were optimized to reduce the computation amount while maintaining the accuracy, thus improving the inference speed; secondly, in order to enhance the detection effect of the model in crowded scenarios, it was decided to introduce the CA, CBAM, and SA attention mechanisms to improve the model’s performance, and the effects on the model before adding the attention mechanism to the last layer of the backbone network and the three re-referentialized paths in the head network were also tested, respectively. Subsequently, the ReD model in DeepSORT was retrained to extract the appearance features of pigs in order to improve the tracking results. Finally, the improved YOLOv7 and the retrained DeepSORT were combined to realize the real-time counting of pigs. The conclusions are as follows:

1. In the process of pig detection, the detection performance of overhead shot angles is better than that of slant shot angles. YOLOv7 achieved improvements of 8.65, 7.53, and 4.25 percentage points in the mAP, recall, and precision of detection in overhead shot angles relative to slant shot angles, which is more favorable for pig detection in a complex environment.
2. Introducing attention mechanisms can further improve the detection accuracy of the model. Among them, the YOLOv7 model with the CA mechanism added in the head network achieved the best comprehensive detection performance. Its mAP, recall, and precision with the overhead shot test dataset were 96.14%, 95.96%, and 97.25%, respectively. In addition, optimizing the ELAN-W module with PConv can significantly reduce the computational complexity of the model. The final improved model achieved respective increases of 3.24, 0.05, and 1.00 percentage points in mAP in slant shots and overhead shots with a simultaneous reduction in computational effort of 3.6 GFLOPS.
3. In the dynamic counting test phase, the original YOLOv7 model combined with DeepSORT achieved counting errors of −4, +5, +19, and −32 with video test sets containing 144, 201, 285, and 294 pigs, respectively, with an average accuracy of 94.33%. After improvement, the YOLOv7 model combined with DeepSORT achieved counting errors of −3, −3, −4, and −26 with the same test sets and an average accuracy of 96.58%. The results show that the improved YOLOv7 has an enhancing effect on the overall counts.

## Figures and Tables

**Table 1:** Distribution of datasets.

Set	Number of Images	Total Number of Pigs	Maximum Number of Pigs in a Single Image	Average Number of Pigs per Image
Train	1441	27,363	30	19
Validation	264	5255	29	19
Test-O	131	3319	30	25
Test-S	133	1540	22	12
Test-All	264	4859	30	18
Total	1969	37,477	28	19

**Table 2:** Parameter settings for YOLOv7 and DeepSORT models.

Model	Parameters	Values
YOLOv7	Image Size	640 × 640
	Batch Size	8
	Learning Rate	0.001
	Epochs	100
	Optimizer	Adam
	weight_decay	0.0005
	Momentum	0.937
DeepSORT	MAX_DIST	0.4
	MIN_CONFIDENCE	0.5
	NMS_MAX_OVERLAP	0.5
	MAX IOU_DISTANCE	0.5
	MAX AGE	70
	N_INIT	3
	NN_BUDGET	100

**Table 3:** YOLOv7 series model performance.

Model	mAP_0.5/%	mAP_0.5:0.95/%	Recall/%	Precision/%	Inference Time/ms	Parameters	Size of Model/MB	Number of Layers
YOLOv7-tiny	85.85	46.41	85.92	94.12	3.12	0.60 ${\times \;10}^{7}$	13	255
YOLOv7	93.57	63.64	93.04	97.16	8.66	3.72 $\times {\;10}^{7}$	71.3	407
YOLOv7x	93.82	63.85	93.52	96.97	12.6	7.08 ${\times \;10}^{7}$	139	459
YOLOv7-w6	91.5	61.0	91.4	97.5	8.91	6.98 ${\times \;10}^{7}$	154	446
YOLOv7-e6	94.20	63.4	94.2	97.15	11.9	9.65 ${\times \;10}^{7}$	188	614
YOLOv7-e6e	90.3	59.7	90.4	96.40	17.60	15.10 ${\times \;10}^{7}$	315	1032
YOLOv7-d6	94.00	63.74	93.95	97.08	13.1	13.29 ${\times \;10}^{7}$	1480	702

**Table 4:** Test results of YOLOv7 after the improvement of different attentional mechanisms on slant shot test datasets, overhead shot test datasets, and all test datasets.

Model	Size of Model/MB	Add Location	Test Set	mAP	Recall/%	Precision/%	Inference Time/ms
YOLOv7	71.3		Test-S	87.51	88.17	93.99	8.66
			Test-O	96.16	95.7	98.24	
			Test-All	93.57	93.04	97.16	
YOLOv7 + CA	71.5	Ⅰ	Test-S	90.34	90.58	95.68	8.64
			Test-O	95.74	95.3	97.92	
			Test-All	94.06	93.7	97.24	
	71.4	Ⅱ	Test-S	91.1	91.17	97.84	8.80
			Test-O	96.14	95.96	97.25	
			Test-All	94.54	94.44	97.41	
YOLOv7 + SA	71.3	Ⅰ	Test-S	88.24	88.7	95.72	8.60
			Test-O	96.16	95.66	98.27	
			Test-All	93.72	93.62	97.33	
	71.3	Ⅱ	Test-S	88.53	89.16	94.76	8.79
			Test-O	95.62	94.96	98.04	
			Test-All	93.45	93.26	96.87	
YOLOv7 + CBAM	71.5	Ⅰ	Test-S	90.33	90.32	93.61	9.24
			Test-O	96.43	96.17	97.97	
			Test-All	94.57	94.26	97.32	
	71.4	Ⅱ	Test-S	91.79	92.4	95.79	9.81
			Test-O	96.41	95.87	98.18	
			Test-All	94.97	94.42	96.93	

**Table 5:** YOLOv7 improved ablation experimental results.

Models	YOLOv7	PConv	CA	Test Set	mAP/%	Recall/%	Precision/%	GFLOPS	Inference Time/ms
Ⅰ	✓			Test-S	87.51	88.17	93.99	103.2	8.66
				Test-O	96.16	95.70	98.24		
				Test-All	93.57	93.04	97.16		
Ⅱ	✓	✓		Test-S	89.00	89.42	94.64	99.5	8.47
				Test-O	95.42	95.14	97.92		
				Test-All	93.42	93.35	96.61		
Ⅲ	✓		✓	Test-S	91.10	91.17	97.84	103.2	8.80
				Test-O	96.14	95.96	97.25		
				Test-All	94.54	94.44	97.41		
Ⅳ	✓	✓	✓	Test-S	90.75	90.71	98.38	99.6	8.55
				Test-O	96.21	95.93	98.24		
				Test-All	94.57	94.38	98.26		

“✓” Indicates that the module is used.

**Table 6:** Improved YOLOv7 with Faster RCNN, YOLOv3, YOLOv4, YOLOv5, and SSD and Test-All test datasets.

Model	Size of Model/MB	mAP/%	Recall/%	Precision/%	FPS
Faster RCNN	521	87.52	91.29	65.33	21.92
YOLOv3	235	92.41	92.71	97.43	35.13
YOLOv4	244	89.37	89.81	96.83	25.23
YOLOv5	40.1	92.5	86.90	95.90	70.55
SSD (mobilenetv2)	14.3	74.84	76.18	94.41	116.05
YOLOv7	71.3	93.57	93.04	97.16	55.97
Improved-YOLOv7	71.3	94.57	94.38	98.26	56.70

**Table 7:** Comparison of counting results of different models and different counting strategies.

Framework	*^2^ video-144		*^2^ video-201		*^2^ video-285		*^2^ video-294		Average Accuracy	FPS
	Errors	Accuracy/%	Errors	Accuracy/%	Errors	Accuracy/%	Errors	Accuracy/%		
YOLOv7 + DeepSORT	+54	62.5	+118	41.3	+229	19.7	+175	41.5	41.25	22
Improved-YOLOv7 + DeepSORT	+52	63.9	+106	47.3	+211	26.0	+148	50.5	46.93	21
*^1^ YOLOv7 + DeepSORT	−4	97.2	+5	97.5	+19	93.3	−32	89.3	94.33	22
*^1^ Improved-YOLOv7 + DeepSORT	−3	97.9	−3	98.5	−4	98.6	−26	91.3	96.58	22

*^1^ The use of the counting strategy in this paper. *^2^ The number after “video-” is the actual number of pigs in the video.

## Data Availability

The data presented in this study are available on request from the corresponding author.

## References

[1] Oh S.H., Whitley N.C. (2011). Pork Production in China, Japan and South Korea. Asian-Australas. J. Anim. Sci..

[2] Yao Y. Multi-Measures to Promote the High-Quality Development of the Hog Industry. http://guoqing.china.com.cn/2024-03/01/content_117030011.htm.

[3] Zhang T., Liang Y., He Z. (2016). Applying image recognition and counting to reserved live pigs statistics. Comput. Appl. Softw..

[4] Idoje G., Dagiuklas T., Iqbal M. (2021). Survey for smart farming technologies: Challenges and issues. Comput. Electr. Eng..

[5] Bao W., Lin Z., Hu G., Liang D., Huang L., Zhang X. (2023). Method for wheat ear counting based on frequency domain decomposition of MSVF-ISCT. Inf. Process. Agric..

[6] Syazwani R.W.N., Asraf H.M., Amin M.M.S., Dalila K.N. (2022). Automated image identification, detection and fruit counting of top-view pineapple crown using machine learning. Alex. Eng. J..

[7] Pandit A., Rangole J., Shastri R., Deosarkar S. Vision system for automatic counting of silkworm eggs. Proceedings of the International Conference on Information Communication and Embedded Systems (ICICES2014).

[8] Yamamoto K., Guo W., Yoshioka Y., Ninomiya S. (2014). On plant detection of intact tomato fruits using image analysis and machine learning methods. Sensors.

[9] He K., Zhang X., Ren S., Sun J. Deep residual learning for image recognition. Proceedings of the IEEE Conference on Computer Vision and Pattern Recognition.

[10] Liu W., Anguelov D., Erhan D., Szegedy C., Reed S., Fu C.-Y., Berg A.C. (2016). Ssd: Single shot multibox detector. Computer Vision–ECCV 2016: 14th European Conference, Amsterdam, The Netherlands, October 11–14, 2016, Proceedings, Part I 14.

[11] Tan M., Pang R., Le Q.V. Efficientdet: Scalable and efficient object detection. Proceedings of the IEEE/CVF Conference on Computer Vision and Pattern Recognition.

[12] Redmon J., Divvala S., Girshick R., Farhadi A. You only look once: Unified, real-time object detection. Proceedings of the IEEE Conference on Computer Vision and Pattern Recognition.

[13] Xiong H., Cao Z., Lu H., Madec S., Liu L., Shen C. (2019). TasselNetv2: In-field counting of wheat spikes with context-augmented local regression networks. Plant Methods.

[14] Wang D., Zhang D., Yang G., Xu B., Luo Y., Yang X. (2021). SSRNet: In-field counting wheat ears using multi-stage convolutional neural network. IEEE Trans. Geosci. Remote Sens..

[15] He L., Fang W., Zhao G., Wu Z., Fu L., Li R., Majeed Y., Dhupia J. (2022). Fruit yield prediction and estimation in orchards: A state-of-the-art comprehensive review for both direct and indirect methods. Comput. Electron. Agric..

[16] Jiang K., Xie T., Yan R., Wen X., Li D., Jiang H., Jiang N., Feng L., Duan X., Wang J. (2022). An attention mechanism-improved YOLOv7 object detection algorithm for hemp duck count estimation. Agriculture.

[17] Xu B., Wang W., Falzon G., Kwan P., Guo L., Chen G., Tait A., Schneider D. (2020). Automated cattle counting using Mask R-CNN in quadcopter vision system. Comput. Electron. Agric..

[18] Wang R., Gao R., Li Q., Feng L., Bai Q., Ma W. (2022). High-density Pig Herd Counting Method Combined with Feature Pyramid and Deformable Convolution. Trans. Chin. Soc. Agric. Mach..

[19] Tian M., Guo H., Chen H., Wang Q., Long C., Ma Y. (2019). Automated pig counting using deep learning. Comput. Electron. Agric..

[20] Feng W., Wang K., Zhou S. (2023). An efficient neural network for pig counting and localization by density map estimation. IEEE Access.

[21] Chen G., Shen S., Wen L., Luo S., Bo L. Efficient pig counting in crowds with keypoints tracking and spatial-aware temporal response filtering. Proceedings of the 2020 IEEE International Conference on Robotics and Automation (ICRA).

[22] Ju M., Choi Y., Seo J., Sa J., Lee S., Chung Y., Park D. (2018). A Kinect-based segmentation of touching-pigs for real-time monitoring. Sensors.

[23] Yang Q., Chen M., Huang Y., Xiao D., Liu Y., Zhou J. (2023). Pig Counting Algorithm Based on Improved YOLO v5n. Trans. Chin. Soc. Agric. Mach..

[24] Hao W., Zhang L., Han M., Zhang K., Li F., Yang G., Liu Z. (2023). YOLOv5-SA-FC: A Novel Pig Detection and Counting Method Based on Shuffle Attention and Focal Complete Intersection over Union. Animals.

[25] Gochoo M., Rizwan S.A., Ghadi Y.Y., Jalal A., Kim K. (2021). A systematic deep learning based overhead tracking and counting system using RGB-D remote cameras. Appl. Sci..

[26] Parico A.I.B., Ahamed T. (2021). Real time pear fruit detection and counting using YOLOv4 models and deep SORT. Sensors.

[27] Cao Y., Chen J., Zhang Z. (2023). A sheep dynamic counting scheme based on the fusion between an improved-sparrow-search YOLOv5x-ECA model and few-shot deepsort algorithm. Comput. Electron. Agric..

[28] Kim J., Suh Y., Lee J., Chae H., Ahn H., Chung Y., Park D. (2022). EmbeddedPigCount: Pig counting with video object detection and tracking on an embedded board. Sensors.

[29] Huang Y., Xiao D., Liu J., Tan Z., Liu K., Chen M. (2023). An Improved Pig Counting Algorithm Based on YOLOv5 and DeepSORT Model. Sensors.

[30] Pig Counting Challenge. https://challenge.xfyun.cn/topic/info?type=pig-check.

[31] Wang C.-Y., Bochkovskiy A., Liao H.-Y.M. YOLOv7: Trainable bag-of-freebies sets new state-of-the-art for real-time object detectors. Proceedings of the IEEE/CVF Conference on Computer Vision and Pattern Recognition.

[32] WongKinYiu YOLOv7. https://github.com/WongKinYiu/yolov7.

[33] Zhang X., Zeng H., Guo S., Zhang L. Efficient long-range attention network for image super-resolution. Proceedings of the European Conference on Computer Vision.

[34] Chen J., Kao S.-h., He H., Zhuo W., Wen S., Lee C.-H., Chan S.-H.G. Run, Don’t Walk: Chasing Higher FLOPS for Faster Neural Networks. Proceedings of the IEEE/CVF Conference on Computer Vision and Pattern Recognition.

[35] Hu J., Shen L., Sun G. Squeeze-and-excitation networks. Proceedings of the IEEE Conference on Computer Vision and Pattern Recognition, Salt Lake City.

[36] Woo S., Park J., Lee J.-Y., Kweon I.S. Cbam: Convolutional block attention module. Proceedings of the European Conference on Computer Vision (ECCV).

[37] Hou Q., Zhou D., Feng J. Coordinate attention for efficient mobile network design. Proceedings of the IEEE/CVF Conference on Computer Vision and Pattern Recognition.

[38] Wojke N., Bewley A., Paulus D. Simple online and realtime tracking with a deep association metric. Proceedings of the 2017 IEEE International Conference on Image Processing (ICIP).

[39] Wu T., Zhong S., Chen H., Geng X. (2023). Research on the Method of Counting Wheat Ears via Video Based on Improved YOLOv7 and DeepSort. Sensors.

[40] Zhang Q.-L., Yang Y.-B. Sa-net: Shuffle attention for deep convolutional neural networks. Proceedings of the ICASSP 2021—2021 IEEE International Conference on Acoustics, Speech and Signal Processing (ICASSP).

[41] Selvaraju R.R., Cogswell M., Das A., Vedantam R., Parikh D., Batra D. Grad-cam: Visual explanations from deep networks via gradient-based localization. Proceedings of the IEEE International Conference on Computer Vision.

[42] Ye M., Shen J., Lin G., Xiang T., Shao L., Hoi S.C. (2021). Deep learning for person re-identification: A survey and outlook. IEEE Trans. Pattern Anal. Mach. Intell..

[43] Liu Z., Sun M., Zhou T., Huang G., Darrell T. (2018). Rethinking the value of network pruning. arXiv.

